# Effects of Dietary Supplementation with Yeast Hydrolysate on Immune Function, Fecal Short Chain Fatty Acids, and Intestinal Health in Cats

**DOI:** 10.3390/vetsci12030239

**Published:** 2025-03-03

**Authors:** Jintao Sun, Shukun Liang, Xinshu Gu, Jie Xu, Xiumin Wang, Zhenlong Wang, Hui Tao, Jinquan Wang, Bing Han

**Affiliations:** 1 Key Laboratory of Feed Biotechnology of Ministry of Agriculture and Rural Affairs, Institute of Feed Research, Chinese Academy of Agricultural Sciences, No. 12 Zhong Guan Cun South Street, Haidian District, Beijing 100081, China; 82101235453@caas.cn (J.S.); 18811536925@163.com (S.L.); 15957544121@163.com (X.G.); wangxiumin@caas.cn (X.W.); wangzhenlong01@caas.cn (Z.W.); taohui@caas.cn (H.T.); wangjinquan@caas.cn (J.W.); 2 School of Veterinary Medicine, China Agricultural University, Beijing 100193, China; 3 Angel Yeast Co., Ltd., 168 Chengdong Avenue, Yichang 443000, China; xujie@angelyeast.com

**Keywords:** yeast hydrolysate, fecal microbiota, cats, 3-methylindole

## Abstract

Yeast hydrolysate (YH) is rich in amino acids and other nutrients, and as a nutritional supplement it has been widely used in daily nutritional supplements for livestock. However, the role of YH in domestic pets, especially cats, has not yet been determined. The objective of this research was to study the effects of different concentrations of YH on the healthy cats. All cats were randomly divided into four treatments: the control group (T0, *n* = 6, without YH), treatment 1 (T1, the low concentration group, 0.8% of YH, *n* = 6), treatment 2 (T2, the middle concentration group, 1.5% of YH, *n* = 6), and treatment 3 (T3, the high concentration group, 4% of YH, *n* = 6), and the blood biochemistry, immune indexes and odorous substances in the feces, and microbiome of cats were determined on day 28. Our results showed that YH could increase the immunoglobulin G (IgG) level in the serum (*p* < 0.01) and reduce 3-methylindole content in the feces (*p* < 0.01). The acetic acid in the feces of T2 treatment (1.5%YH) was apparently increased compared to the control treatment (*p* < 0.05). The blood biochemistry indexes were not affected by the YH. Compared to the control group, there was no significant difference in the abundance at the phylum level. On the genus level, the abundance of *g*_*Ruminocococcaceae* and *g_Lachnospiraceae*, the beneficial bacteria in the gut, were decreased in the treatment T3 compared to treatment T1 (*p* < 0.05), but there was no significance between T1 and T2, which suggested that a high concentration of YH may be negative for gut health. So, the research showed that 1.5% of YH could be the best concentration for the improvement of immunity and gut health for cats.

## Simple Summary

The research focused on the effects of yeast hydrolysate on serum chemistry and gut health in cats. The results indicated that yeast hydrolysate could serve as a beneficial functional additive for pet health.

## 1. Introduction

Different types of yeast, including yeast cell walls, yeast hydrolysate, yeast extract, yeast culture, oligosaccharide, and so on are widely used in livestock feed, and have attracted attention for their potential use in pet food in recent years. Some yeast-related products have showed a benefit for pets for their functional ingredients, especially in dogs [1,2,3,4,5].

Yeast hydrolysate (YH) is a kind of hydrolysate from yeast cells, and has been widely used in feed additions in the livestock industry. YH is mainly derived from yeast (e.g., *Saccharomyces cerevisiae*) and obtained by hydrolyzing yeast cells with specific enzymes, which contains a large abundance of nutrients, including B vitamins, amino acids, nucleotides, β-glucan and mannan oligosaccharides, small peptides, different kinds of enzyme, and other important growth factors, as well as other rich functional substances [6]. These nutrients could promote growth, enhance immunity, improve antioxidant capacity, maintain intestinal health, perform other biological functions, and, furthermore, improve the animal welfare of piglets [7,8]. Adding yeast to feed can also improve the palatability of feed and promote nutrient absorption in broilers [9]. These studies showed the real benefit of yeast on livestock animals.

There are more and more studies focusing on the application of yeast in canine nutrition in recent years. Live yeast could be beneficial for the gut health of dogs by increasing the abundance of *Bifidobacterium* and butyrate in the feces [10]. Studies have shown that yeast cell walls could also improve intestinal health during an abrupt diet transition process [11]. β-glucan from yeast was indicated to prevent obesity in dogs [12], but now there is still a lack of comprehensive studies on the effects of yeast hydrolysate in healthy cats. Therefore, the purpose of this study was to determine the functions of YH by testing blood biochemical indexes, blood immune indexes, fecal metabolites, and fecal microbiota in healthy cats. The research will provide a theoretical basis for the better use of YH as a diet supplement for cats in the future.

## 2. Materials and Methods

### 2.1. Experimental Animals and Sample Collection

The animal experiment was implemented according to the Animal Care and Use Committee of the Institute of Feed Research of the Chinese Academy of Agricultural Sciences (CAAS), and was approved by the Laboratory Animal Ethical Committee and its inspection of the Institute of Feed Research of CAAS (IFR-CAAS-20220801).

**Figure 1:**
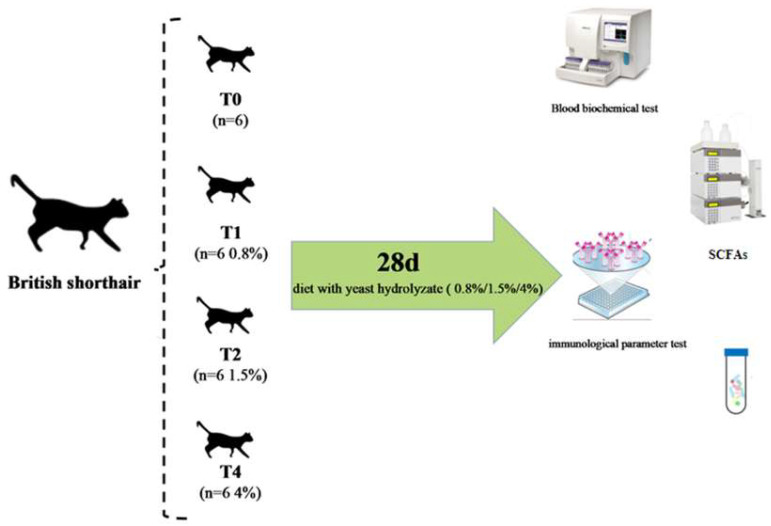
The design of analysis.

Twenty-four healthy adult cats (all about 2.5 years old, female, intact, 3.15 ± 0.59 kg, British shorthair) were raised on the pilot base of the CAAS. Each cat was in an independent space, and the cat housing stainless steel cages in the research were 162 × 68 × 189 cm^3^ and also equipped with a bed, a cat litter tray, and two bowls for feeding and water. The test was carried out at 55.10% humidity. After one week of adaptation, all cats were randomly divided into four treatments: the control group (T0, n = 6, without any yeast product), treatment 1 (T1, the low concentration group, 0.8%, n = 6, 0.26 g/kg body weight on average), treatment 2 (T2, the middle concentration group, 1.5%, n = 6, 0.50 g/kg body weight on average), and treatment 3 (T3, the high concentration group, 4%, n = 6, 1.33 g/kg body weight on average) (Figure 1). The YH product (Dry Matter = 95.99%, Crude Protein = 51.26%, Crude Fat = 3.02%, Crude Fibre = 0.1%, Crude Ash = 6.57%; Glucan 14.43%, Mannan oligosaccharide 11.86%) was provided by Angel Yeast Corporation, Limited (Yichang, Hubei, China). In the analysis, every cage was equipped with food bowls, also, clean water was applied at any time. Animals were fed once at the same time every day, with 100 g feed. The body weight and feed intake of each cat was recorded in the analysis. The temperature of the room was kept at 25 ± 2 °C.

The feed formula was designed referring to the standard of the Association of American Feed Control Officials (AAFCO, 2021), and was shown in Table 1. The nutrient parameters of the feed, including crude protein, crude fat, crude ash, dry matter, and the content of calcium (Ca) and phosphorus (P) were tested on the basis as-is.

On the 28th day, blood samples were collected from the hind legs of cats, and stored at −20 °C for detection of blood indexes and immunological indexes, and when the blood was collected the animals were held with a towel [13]. Fresh fecal samples were collected within one hour; one part of the fecal sample was stored at −20 °C for the detection of short-chain fatty acid (SCFA) and fecal odor, and the other part of the fecal sample was stored at −80 °C for the detection of microbial diversity. Antibiotics or other probiotics were not used in the study.

### 2.2. Blood Biochemical Test

On the 28th day, blood samples from the cats were collected in EDTA tubes without anticoagulants. The collected non-anticoagulated blood was centrifuged at 1200× *g* for 15 min and stored at 4 °C. After centrifugation, the serum was separated for detection. Biochemical indicators of blood supernatant, including total protein (TP), albumin (ALB), globulin (GLO), triglyceride (TG), cholesterol (CHOL), total bilirubin (TBIL), alanine aminotransferase (ALT), aspartate aminotransferase (AST), γ-glutamyl transpeptidase (GGT), Alkaline Phosphatase (ALP), total bile acid (TBA), amylase (AMY), glucose (GLU), creatinine (CRE), and blood urea nitrogen (BUN) were all detected by a biochemical analyzer (MNCHIP Technology Co., Ltd., Tianjin, China). The analysis was implemented according to manufacturer’s instructions.

### 2.3. Analysis of Immunological Parameter of Blood

The kit assay (Jiangsu Meimian Industrial Co., Ltd., Yancheng, Jiangsu, China) was used to test the immunoglobulin A (IgA), immunoglobulin G (IgG), and immunoglobulin M (IgM) of the blood on day 28 by the ELISA method.

### 2.4. Analysis of Short Chain Fatty Acid (SCFA) Volume of the Feces

On the 28th day, fresh feces samples were collected and stored at −20 °C, and the contents of SCFA in each fecal sample were detected separately. Fecal samples from four groups were tested by ion chromatography (Metrohm, Herisau, Switzerland). Fecal samples weighing 0.5 g were taken from each group and dissolved in 200 µL of ZnSO_4_ (144 g/L) and 100 µL of K_4_Fe(CN)_6_ 3H_2_O, and the liquid was diluted to 5 mL. The solution was centrifuged at 7379× *g* for 10 min, and 1 mL of supernatant was used to dilute to 5 mL with water, and the contents of SCFA were tested by ion chromatography referred above. The type of chromatographic column was Metrosep Organic Acids-250/7.8 61005200, and the mobile phase was 0.5 mM H_2_SO_4_, with the flow rate 0.8 mL/min.

### 2.5. Analysis of the Benzpyrole and 3-Methylindole of the Feces

Fresh fecal samples were collected on the 28th day, and 2-g fecal samples were taken from each group and extracted in 10 mL methanol, then washed in water at 40 °C for 20 min. The supernatant of each sample was centrifuged at 10,625× *g* for 10 min, and the benzpyrole and 3-methylindole were tested by High Performance Liquid Chromatography (HPLC) (SHIMADZU, Kyoto, Japan). The mobile phase was 60% acetonitrile, with the flow rate 1 mL/min. Benzpyrole and 3-methylindole were simultaneously detected at 263 nm.

### 2.6. Extraction of Fecal DNA

Microbial genomic DNA was extracted from 24 fecal samples and tested for concentration separately using a E.Z.N.A Mag-Bind Soil DNA Kit (Omega, M5635-02, Irving, TX, USA) and Quibit dsDNA HS (Thermo, Waltham, MA, USA). The extracted samples were stored at −80 °C and used for the next application.

### 2.7. PCR Amplication

The forward primer sequence was CCTACGGGNGGCWGCAG, and the reverse primer was GACTACHVGGGTATCTAATCC [14]. The PCR reaction conditions were as follows: 94 °C, 3 min, 5 cycles at 94 °C, 30 s, 45 °C 20 s, 65 °C 30 s; 20 cycles at 94 °C 20 s, 55 °C 20 s, 72 °C, 30 s; 72 °C 5 min. PCR products were purified and quantified by QIAquick gel extraction kit (QIAGEN, Hilden, Germany) and Quant-iT PicoGreen dsDNA assay kit (Life Technologies, Carlsbad, CA, USA), respectively. The extracted DNA samples were sequenced for 16S rDNA in Sangon Biotech (Shanghai, China) Co., Ltd.

### 2.8. Data Analysis

Gut microbiota evaluation was made using QIIME2 and the R package 3.5.1, whereas alpha diversity indices and the bacterial abundance data of different groups were compared by performing the Kruskal–Wallis test followed by the pairwise Mann–Whitney U comparison. Then, the resulting *p*-values were corrected with the Bonferroni method. The alpha diversity analysis was conducted to investigate the microbial diversity using the Shannon diagram, and β-diversity analysis was conducted to investigate the structural variation of microbial communities across samples using Bray–Curtis and visualized via principal coordinate analysis (PCoA).

Except the 16S rRNA data described in detail, other data obtained in this study were first verified for homogeneity of variances by Levene test, and then analyzed with a one-way ANOVA test followed by Tukey’s multiple range test; data were expressed as the mean ± SEM by Tukey’s multiple range test, and it should be noted that a *p*-value of *p* < 0.05 was considered statistically significant. The following *p*-values were used: * *p* < 0.05, ** *p* < 0.01. These related statistical analyses were performed using SPSS 25.0 (SPSS Inc., Chicago, IL, USA) software.

## 3. Results

### 3.1. Blood Biochemistry

As shown in Table 2, after 28 days of YH feeding, there were no significant changes in blood biochemistry indexes (*p* > 0.05).

### 3.2. Immune Defense Ability

**Figure 2:**
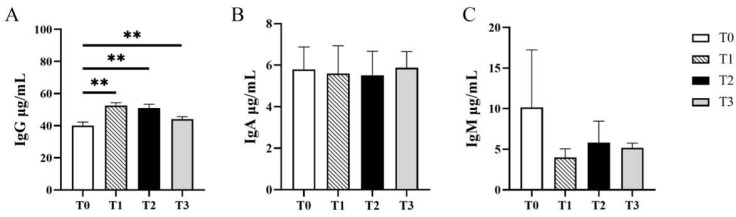
Changes of immunoglobulin in serum of different concentrations of YH. (**A**) IgG content in cat serum on day 28. (**B**) IgA content in cat serum on day 28. (**C**) IgM content in cat serum on day 28. ** *p* < 0.01. *p* > 0.05 meant no significance and was not marked on the figure.

After feeding YH for 28 days, the contents of immunoglobulins, including IgA, IgG and IgM in the serum, were separately detected by ELISA. Compared with T0 group, it was found that the serum IgG of cats fed with YH in treatment T1, T2 and T3 were all increased significantly (*p* < 0.01), but there was no significance among the treatments using YH (*p* > 0.05). But the changes of IgA and IgM in the serum in different treatments were not significant (*p* > 0.05) (Figure 2).

### 3.3. Short Chain Fatty Acid (SCFA)

On day 28, six kinds of SCFA in fecal samples were detected by ion chromatography (Metrohm, Herisau, Switzerland). Compared with T0 and T3, the contents of acetic acid in the T2 treatment were increased significantly (*p* < 0.05). Although, there was no significant difference in the contents of other SCFAs in treatment T1 and T3 compared to T0 (*p* > 0.05), the contents of other SCFAs of T2 were the highest. It is suggested that a certain concentration of YH may affect metabolism and increase the productions of SCFA (Table 3).

### 3.4. Indoles and 3-Methylindole

**Figure 3:**
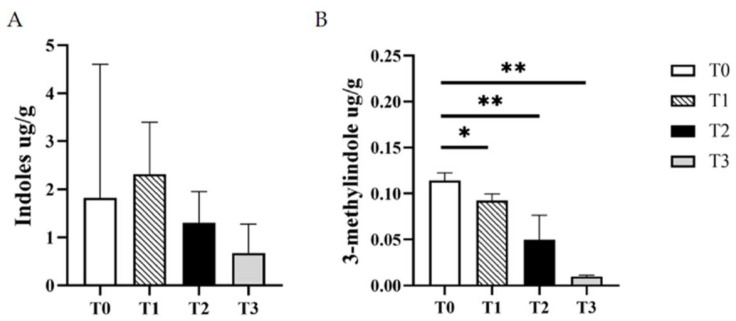
Changes of fecal metabolites of different concentrations of YH. (**A**) Contents of indoles in the cat’s feces on day 28; (**B**) Contents of 3-methylindole in the cat’s feces on day 28. * *p* < 0.05, ** *p* < 0.01.

After feeding YH for 28 days, the contents of indoles and 3-methylindole in cat feces were detected by high-performance liquid chromatography (HPLC). Compared with the T0 group, the 3-methylindole content in feces of T1, T2, and T3 were all decreased significantly (*p* < 0.01), especially in T3 (Figure 3B). The indole content did not change significantly (*p* > 0.05) (Figure 3A). It was suggested that YH could reduce the concentration of 3-methylindole in the feces of cats.

### 3.5. Microbiota in the Feces

**Figure 4:**
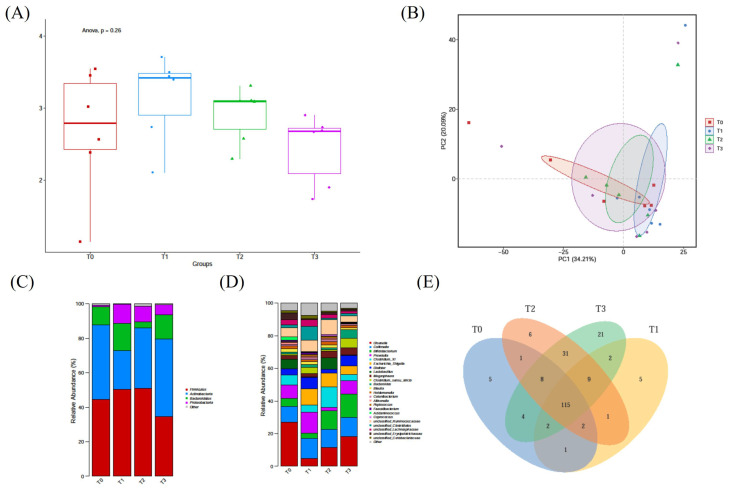
The effect of YH on the richness, α-diversity and β-diversity of fecal microbiota on the phylum level in cats. (**A**) Shannon indices. (**B**) Principal coordinate analysis (PCoA). (**C**) The phylum level of different treatments. (**D**) The genus level of different treatments. (**E**) Venn diagram of different treatments.

There was no significance among different treatments of α-diversity of microbiota (*p* > 0.05) (Figure 4A). In order to detect the difference between microbial communities, the principal coordinate analysis was used to calculate β-diversity, which showed Bray–Curtis dissimilarity. The PCoA diagram were showed in Figure 4B, which showed no significance among the four treatments of β-diversity of microbiota, and showed YH may not affect the diversity and whole abundance of microbiota. Figure 4C showed the abundance of microbiota on the phylum level, which showed the composition of microbiota was changed to some extent.

On the genus level, it can be seen that the abundance of *g*_*Ruminococcaceae* and *g_Lachnospiraceae* in the T3 group were decreased significantly compared to the T1 treatment (*p* < 0.05)

## 4. Discussion

As one of the protein sources, YH contains many functional contents, like yeast polysaccharide, which could enhance the immune function of animals, weaken the inflammatory reaction of animals, and alleviate the occurrence of common nutritional metabolic diseases, such as obesity and malnutrition, so as to reduce the probability of secondary diseases and improve animal welfare [15,16,17,18]. In recent years, most studies about YH in dogs raised, most of them showed the positive effects on the immunity [19,20], fecal microbiota [21], nutrient digestibility [22], and even oxidative stress [23] of dogs. The recent studies about yeast cell walls in cats showed that they could be effective for cats by modulating fecal microbiota in cats, the reason of which was probably for the abundance of oligosaccharide as prebiotics for cats [24,25], although the study of YH in cats has been very limited.

Gut microbiota was important for pets, which was closely related with the gut health and immunity of animals. In our research, although the diversity and the abundance of the microbiota with YH was not significant compared to control, the abundance of Firmicutes was increased in the 0.8% and 1.5% YH treatments, while decreased in the 4.0% YH treatment. Corresponding to this, the abundance of *g_Ruminocococcaceae* and *g_Lachnospiraceae* in the 4.0% YH treatment was decreased significantly compared to 0.8% and 1.5% concentration, and the two genus was closely related with the producing of SCFAs [26,27], including acetic, propiopic and butyric acids, which was important for gut health by fuelling gut epithelial cells, modulating gut pH, playing as the intestinal barrier and other important functions [28,29]. In the treatment of 1.5% concentration, the concentration of acetic acid was apparently significantly higher than other treatments (*p* < 0.05), with other SCFAs higher but without significance, and the former study has proved yeast cell wall could increase SCFAs [30], mainly formic acid and butyric acid. The mechanism for yeast promoting the production of SCFAs was that the probiotics could probably promote the growth of the microbiota related with the carbohydrate digestibility.

Odorous substances in the feces could reflect the digestibility of nutrients in feed to some extent. In this research, the content of 3-methylindole in cat feces was reduced significantly. The reason was probably that the YH was more easily digestible for being rich in peptides [31], which was also recorded in the former study [22], also showing the dried whole-cell yeast could be easily digestible.

In addition to the above, gut health was closely connected with the immunity of animals [32]. Studies have shown that polysaccharide from yeast could promote animal immunity and accelerate the maturation of immune function [33]. In this study, it was found that the serum IgG of cats fed with YH was increased significantly (*p* < 0.01), which was proven by previous studies of other animals [34]. However, our study just focused on the contents of immunoglobulin and did not involve other related factor, which should be considered to verify the effects on the immune system.

To date, the studies of YH have focused more on livestock, and this study supplements these by focusing on the yeast product in cats.

## 5. Conclusions

The YH of different concentrations had a positive effect on immunity and gut health by increasing the IgG content in serum, reducing 3-methylindole content in feces, and increasing the acetic acid in the feces, while a high concentration of YH could reduce the abundance of beneficial bacterium, including *g*_*Ruminocococcaceae* and *g_Lachnospiraceae*, which showed 1.5% could be the appropriate concentration for cats. In future, YH could be a beneficial functional additive for pet health.

## Figures and Tables

**Table 1:** The formula of the cat feed.

Constitutents of Feed, %		Different Treatments			
		T0	T1	T2	T3
Material proportion	Corn,%	29.94	29.70	29.48	29.32
	Rice, %	17.96	17.82	17.69	17.59
	Chicken powder,%	29.94	29.70	29.48	27.36
	Duck meal, %	10.98	10.89	10.81	10.75
	Meat and bone meal, %	4.99	4.95	4.91	4.89
	Beet pulp, %	4.99	4.95	4.91	4.89
	YH, %	0.00	0.80	1.50	4.00
	Taurine, %	0.20	0.20	0.20	0.20
	1% Premix,%	1.00	1.00	1.00	1.00
	total	100.00	100.00	100.00	100.00
Nutrients proportion (wet matter calculated)	Crude protein,%	29.91	29.50	31.52	31.61
	Crude fat,%	12.39	12.80	12.76	12.57
	Crude ash,%	7.78	7.58	8.02	7.75
	Dry matter, %	93.69	92.04	94.12	93.74
	Ca, %	1.30	1.30	1.30	1.30
	P, %	0.90	0.90	0.90	0.90

**Table 2:** The blood biochemical indexes of cat.

Index	T0	T1	T2	T3	Reference Range	*p*-Value
TP, g/L	80.52 ± 2.40	82.93 ± 4.65	77.53 ± 3.47	71.74 ± 9.82	54–89	0.527
ALB, g/L	30.37 ± 1.22	30.85 ± 2.23	29.92 ± 1.93	28.78 ± 2.19	22–45	0.896
GLO, g/L	50.15 ± 1.70	52.08 ± 3.75	47.62 ± 2.83	37.28 ± 6.80	15–57	0.077
A/G	0.62 ± 0.03	0.60 ± 0.07	0.62 ± 0.06	0.70 ± 0.09	-	0.724
TBIL, µmol	6.07 ± 1.12	5.31 ± 0.89	3.87 ± 0.36	6.24 ± 1.62	2–15	0.375
ALT, U/L	55.67 ± 9.56	63.33 ± 4.45	61.33 ± 4.88	47.67 ± 7.97	8.2–123	0.536
AST, U/L	39.00 ± 5.22	38.50 ± 2.95	28.50 ± 3.40	32.20 ± 6.51	9.2–39.5	0.310
AST/ALT	0.81 ± 0.18	0.61 ± 0.04	0.48 ± 0.06	0.42 ± 0.05	-	0.063
GGT, U/L	1.18 ± 0.12	1.00 ± 0.19	0.85 ± 0.25	0.64 ± 0.16	0–2	0.266
ALP, U/L	26.50 ± 4.40	19.17 ± 2.95	21.67 ± 2.50	21.20 ± 2.27	10–90	0.429
TBA, µmol	2.17 ± 0.28	2.74 ± 0.51	3.36 ± 0.74	3.67 ± 1.02	0–15	0.411
AMY, U/L	1984.50 ± 279.44	1730.83 ± 161.81	1555.50 ± 223.75	1320.60 ± 202.15	400–3000	0.243
TG, mmol	0.65 ± 0.11	0.62 ± 0.04	0.61 ± 0.05	0.55 ± 0.08	0.1–0.9	0.841
CHOL, mmol	3.27 ± 0.35	3.31 ± 0.46	2.80 ± 0.19	3.34 ± 0.49	1.68–5.81	0.710
GLU, mmol	4.67 ± 0.13	5.07 ± 0.37	5.30 ± 0.32	5.34 ± 0.55	4.11–8.84	0.543
CRE, µmol	93.67 ± 8.07	98.83 ± 14.07	81.67 ± 11.24	100.4 ± 14.59	27–223	0.689
BUN, mmol	7.33 ± 0.35	7.51 ± 0.54	7.14 ± 0.53	8.73 ± 0.88	3.6–15.5	0.269

Notes: mean ± SEM; SEM, standard error of the mean. Abbreviations: TP, total protein; ALB, albumin; GLO, globulin; TG, triglyceride; CHOL, cholesterol; TBIL, total bilirubin; ALT, alanine aminotransferase; AST, aspartate aminotransferase; GGT, γ-glutamyl transpeptidase; ALP, Alkaline Phosphatase; TBA, total bile acid; AMY, amylase; GLU, glucose; CRE, creatinine; BUN, blood urea nitrogen.

**Table 3:** Contents of SCFA in feces.

Treatments (g/dL)	Acetic Acid	Propionic Acid	Butyric Acid	Isobutyric Acid	Isovaleric Acid	Valeric Acid
T0	0.546 ± 0.031 ^b^	0.222 ± 0.028	0.221 ± 0.061	0.031 ± 0.013	0.050 ± 0.019	0.097 ± 0.018
T1	0.699 ± 0.097 ^ab^	0.313 ± 0.039	0.162 ± 0.030	0.032 ± 0.009	0.052 ± 0.007	0.060 ± 0.017
T2	0.825 ± 0.061 ^a^	0.357 ± 0.047	0.277 ± 0.030	0.035 ± 0.009	0.059 ± 0.007	0.138 ± 0.021
T3	0.678 ± 0.047 ^b^	0.314 ± 0.071	0.185 ± 0.039	0.028 ± 0.005	0.033 ± 0.008	0.084 ± 0.014

Notes: Different upper right lowercase meant the significance (*p* < 0.05). Contents of six short-chain fatty acids (acetic acid, propionic acid, isomeric acid, butyric acid, isomeric acid, valeric acid) in the cat’s feces on day 28.

## Data Availability

The data presented in this study are available upon request from the corresponding author.
